# Effect of Neuraminidase Inhibitor–Resistant Mutations on Pathogenicity of Clade 2.2 A/Turkey/15/06 (H5N1) Influenza Virus in Ferrets

**DOI:** 10.1371/journal.ppat.1000933

**Published:** 2010-05-27

**Authors:** Natalia A. Ilyushina, Jon P. Seiler, Jerold E. Rehg, Robert G. Webster, Elena A. Govorkova

**Affiliations:** 1 Division of Virology, Department of Infectious Diseases, St. Jude Children’s Research Hospital, Memphis, Tennessee, United States of America; 2 Laboratory of Virus Physiology, The D.I. Ivanovsky Institute of Virology RAMS, Moscow, Russia; 3 Department of Pathology, St. Jude Children’s Research Hospital, Memphis, Tennessee, United States of America; 4 Department of Pathology, University of Tennessee Health Science Center, Memphis, Tennessee, United States of America; Erasmus Medical Center, Netherlands

## Abstract

The acquisition of neuraminidase (NA) inhibitor resistance by H5N1 influenza viruses has serious clinical implications, as this class of drugs can be an essential component of pandemic control measures. The continuous evolution of the highly pathogenic H5N1 influenza viruses results in the emergence of natural NA gene variations whose impact on viral fitness and NA inhibitor susceptibility are poorly defined. We generated seven genetically stable recombinant clade 2.2 A/Turkey/15/06-like (H5N1) influenza viruses carrying NA mutations located either in the framework residues (E119A, H274Y, N294S) or in close proximity to the NA enzyme active site (V116A, I117V, K150N, Y252H). NA enzyme inhibition assays showed that NA mutations at positions 116, 117, 274, and 294 reduced susceptibility to oseltamivir carboxylate (IC_50_s increased 5- to 940-fold). Importantly, the E119A NA mutation (previously reported to confer resistance in the N2 NA subtype) was stable in the clade 2.2 H5N1 virus background and induced cross-resistance to oseltamivir carboxylate and zanamivir. We demonstrated that Y252H NA mutation contributed for decreased susceptibility of clade 2.2 H5N1 viruses to oseltamivir carboxylate as compared to clade 1 viruses. The enzyme kinetic parameters (V*_max_*, K*_m_* and K*_i_*) of the avian-like N1 NA glycoproteins were highly consistent with their IC_50_ values. None of the recombinant H5N1 viruses had attenuated virulence in ferrets inoculated with 10^6^ EID_50_ dose. Most infected ferrets showed mild clinical disease signs that differed in duration. However, H5N1 viruses carrying the E119A or the N294S NA mutation were lethal to 1 of 3 inoculated animals and were associated with significantly higher virus titers (*P*<0.01) and inflammation in the lungs compared to the wild-type virus. Our results suggest that highly pathogenic H5N1 variants carrying mutations within the NA active site that decrease susceptibility to NA inhibitors may possess increased virulence in mammalian hosts compared to drug-sensitive viruses. There is a need for novel anti-influenza drugs that target different virus/host factors and can limit the emergence of resistance.

## Author Summary

Highly pathogenic avian H5N1 influenza viruses remain a potential pandemic threat. If vaccination is not available in the event of a pandemic, antiviral drugs such as neuraminidase (NA) inhibitors (oseltamivir and zanamivir) will be crucial for disease control. However, the emergence of NA inhibitor–resistant virus mutations can significantly limit the effectiveness of treatment. We used reverse genetics techniques to study the impact of specific point mutations in the NA gene on the pathogenicity of clade 2.2 H5N1 influenza viruses in a ferret animal model. Seven recombinant H5N1 viruses (containing the mutations V116A, I117V, E119A, K150N, Y252H, H274Y, and N294S) were viable and genetically stable in MDCK cells. Three of the NA mutations were of particular significance: (1) E119A conferred resistance to both oseltamivir carboxylate and zanamivir and increased virus virulence in ferrets; (2) H274Y conferred a high level of resistance to oseltamivir carboxylate; and (3) N294S conferred moderate level of resistance to oseltamivir carboxylate and increased virus virulence in ferrets. Strategies to limit the emergence of drug resistance are urgently needed.

## Introduction

The highly pathogenic avian H5N1 influenza viruses remain of concern as a pandemic threat. Of the 486 cases of human H5N1 infection confirmed between March 2003 and March 2010, ∼60% were lethal [1]. The H5N1 influenza viruses have continuously evolved and increased in genetic diversity: to date, 10 H5 hemagglutinin (HA) clades have been distinguished, some of which are geographically widespread [2]. The most epidemiologically significant clades are 1, 2 (subclades 2.1, 2.2, 2.3), and 7 [3].

Although vaccination remains the primary method of influenza control, effective antiviral drugs are the best option when vaccine availability or efficacy is limited [4]. The anti-influenza drugs, neuraminidase (NA) inhibitors (oseltamivir and zanamivir) [5], are sialic acid analogues that selectively target the NA enzyme of influenza A and B viruses [6], [7]. Both are safe and effective for the prophylaxis and treatment of seasonal H1N1 and H3N2 influenza [5]. There is limited information about the clinical use of NA inhibitors against highly pathogenic H5N1 influenza viruses; however, studies in animal models suggest their efficacy [8]–[10]. The orally administered NA inhibitor oseltamivir was chosen for stockpiling for the national pandemic preparedness plan.

In the event of a pandemic, the effectiveness of therapeutic and prophylactic oseltamivir will depend not only on the correct dosage and duration of treatment but also on the susceptibility of the targeted virus strain. Our group [9], [10] and others [11] have shown that the NA inhibitor susceptibility of H5N1 viruses of clades 1 and 2.2 may differ. We found an 18-fold difference between the *in vitro* oseltamivir carboxylate (the active methabolite of oseltamivir) susceptibility of A/Turkey/15/06 (H5N1) virus (clade 2.2) and A/Vietnam/1203/04 (H5N1) virus (clade 1) and different treatment efficacy in mice inoculated with these viruses (20% vs. 80% survival on the same regimen) [8], [9]. Recent data showed that previously undescribed drift NA mutations may also decrease the *in vitro* susceptibility of H5N1 influenza viruses to oseltamivir carboxylate [10]–[13], possibly reducing the efficacy of the drug *in vivo*. These findings demonstrate the need for systematic evaluation of the impact of natural NA gene variations on anti-NA drug susceptibility and on other biological properties of H5N1 influenza viruses.

Oseltamivir and zanamivir were designed to bind only to highly conserved residues within the active site of influenza A and B virus NA protein [6]. This active site comprises 8 functional residues (R118, D151, R152, R224, E276, R292, R371, and Y406; N2 numbering here and throughout) and 11 framework residues (E119, R156, W178, S179, D198, I222, E227, H274, E277, N294, and E425) [14]. Despite a high degree of conservation of these residues, the NA substitutions identified in NA inhibitor-resistant influenza viruses isolated both *in vitro* and clinically tend to be NA subtype–specific: E119A/G/D/V, R292K, and N294S in the N2 and N9 subtypes and H274Y and N294S in the N1 subtype [14], [15]. Broad screening of the *in vitro* susceptibility of seasonal and H5N1 influenza viruses to NA inhibitors together with recent crystal structure data and conformational studies of influenza N1 enzyme identified several additional conserved or semiconserved NA residues (e.g., V116, I117, Q136, K150, D151, and I222) that may also confer resistance [12], [16]–[19]. Importantly, the exact mechanism by which these changes affect susceptibility to a particular NA inhibitor are not yet understood.

Early studies suggested that seasonal influenza viruses resistant to NA inhibitors may be less infective and transmissible in ferrets than their wild-type counterparts [20]–[22]. The two available reports on the fitness of highly pathogenic oseltamivir-resistant H5N1 viruses of clade 1 offered different findings [23], [24]. In ferrets, an oseltamivir-resistant H5N1 virus carrying an H274Y NA mutation replicated approximately 10 times less efficiently in the upper respiratory tract than the wild-type virus [23]. In contrast, neither the H274Y nor the N294S NA mutation compromised the lethality or virulence of clade 1 A/Vietnam/1203/04 (H5N1) virus in mice [24]. This difference in fitness may reflect a difference in virulence, although the question remains to be answered.

In the homogeneous clade 2.2 A/Turkey/15/06-like (H5N1) genetic background, we studied the role of single point NA mutations near or within the enzyme active site on NA inhibitor susceptibility, NA enzyme kinetics, viability, genetic stability, and pathogenesis in ferrets. Seven substitutions were stable in the N1 NA protein and five reduced virus susceptibility to oseltamivir carboxylate or to both NA inhibitors. Infection of ferrets with the recombinant H5N1 viruses caused mild disease of various duration, although NA inhibitor-resistant variants with the E119A and N294S mutations were more virulent than the wild-type virus.

## Results

### Generation, Growth, and Genetic Stability of Recombinant H5N1 Viruses

**Figure 1:**
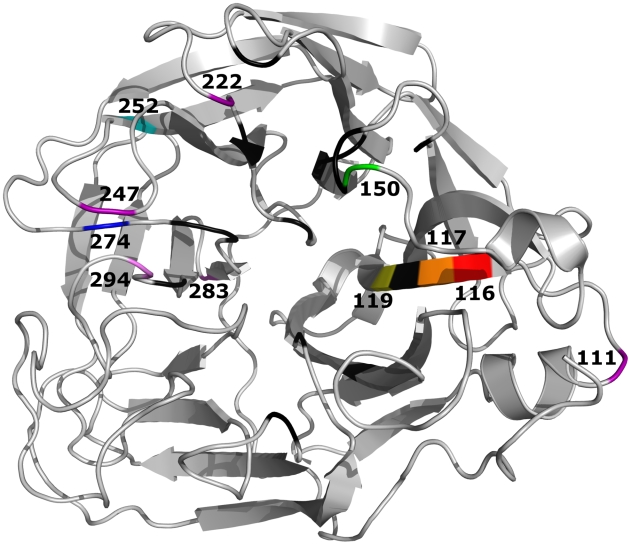
Crystal structure of the A/Vietnam/1203/04 (H5N1) NA molecule (Protein Data Bank:2HTY). Shown are 11 residues in or near the enzyme’s active site (black) that were substituted in this study. Residues shown in purple are positions where a single amino acid substitution affects the stability of the recombinant A/Turkey/15/06-like (H5N1) influenza virus.

We used the eight-plasmid reverse genetics technique to generate 11 recombinant A/Turkey/15/06-like (H5N1) viruses carrying different NA mutations (Figure 1), that were proposed to affect virus susceptibility to NA inhibitors [12], [16]–[19]. Two NA mutations (H274Y and N294S) were selected based on case reports on the isolation of oseltamivir-resistant variants in H5N1 virus infected patients after treatment with oseltamivir [23], [25] or before administration of the drug [26]. Four NA residues (R111, S247, Y252, and D283) were chosen based on the differences of amino acid alignments of the NA active sites of A/Vietnam/1203/04 (H5N1) virus (clade 1) and A/Turkey/15/06 (H5N1) virus (clade 2.2) (data not shown). Five NA residues (V116, I117, E119, K150, and I222) were selected based on the results of NA enzyme inhibition assays that substitutions at these positions may be linked to reduced drug-susceptibility in avian and human viruses carrying N1 NA [19]. The viability of the recombinant viruses was evaluated by rescue from transfected 293T cells. Viruses with the R111K, I222L, S247N, and D283N NA amino acid substitutions could not be rescued in three independent experiments, clearly indicating that these mutations are not stably maintained in the clade 2.2 A/Turkey/15/06-virus background.

The recombinant wild-type (WT) H5N1 influenza virus and seven mutants (V116A, I117V, E119A, K150N, Y252H, H274Y, and N294S) were successfully rescued. Direct sequencing of their HA and NA genes revealed that all mutations were correctly incorporated and no additional changes were present in either gene (data not shown). All of the viruses grew to comparable titers and formed homogeneous plaques in MDCK cells (diameter, 0.3 to 1.7 mm), although viruses with the Y252H, H274Y, and N294S NA mutations formed significantly smaller plaques than did the WT virus (*P*<0.01) (Table 1).

**Table 1:** Growth of Recombinant H5N1 Influenza Viruses Before and After Passaging in MDCK Cells.

H5N1 recombinant virus^a^	Before passaging		3^rd^ passage^b^	
	Virus yield (log_10_PFU/ml)	Plaque size (mm ± SD)	Virus yield (log_10_PFU/ml)	Plaque size (mm ± SD)
**WT**	6.5±0.4	1.6±0.4	6.8±0.3	1.5±0.3
**V116A**	6.2±0.4	1.7±0.4	6.2±0.2	1.8±0.3
**I117V**	6.2±0.2	1.6±0.2	6.4±0.3	1.5±0.2
**E119A**	6.2±0.3	1.4±0.3	6.2±0.4	1.7±0.3
**K150N**	6.3±0.3	1.1±0.3	6.2±0.4	1.1±0.5
**Y252H**	6.3±0.3	0.8±0.3*	6.6±0.8	2.0±0.4°
**H274Y**	6.9±0.2	0.3±0.1*	6.8±0.5	2.7±0.6*,°
**N294S**	6.6±0.3	0.8±0.2*	6.7±0.3	0.5±0.2*

a Amino acid numbering is based on N2 NA [43].

b MDCK cells were infected with recombinant viruses at an MOI of 0.001 PFU/ml and virus yield was determined after 72 h incubation at 37°C. Plaque size of any 10 plaques was measured by the Finescale® comparator (Los Angeles, CA, USA). The HA and NA genes were sequenced after the 3^rd^ passage. The emergence of different subpopulations indicated genetic instability.

* *P*<0.01 compared to WT virus from the same passage (one-way ANOVA).

° *P*<0.01 compared to plaque size after the first passage (unpaired two-tailed *t-*test).

To assay the growth and genetic stability of the recombinant viruses *in vitro*, we serially passaged each virus three times in MDCK cells (Table 1). Virus yields before passaging and after the third passage did not differ significantly. All mutants were able to maintain their plaque phenotype with the exception of those carrying mutations at positions 252 and 274: homogenous large plaques were observed for both Y252H and H274Y viruses after the third passage (Table 1). Sequence analysis after the third passage showed that neither WT virus nor viruses carrying NA mutations at residues 116, 117, 119, 150, 252, 274, and 294 had acquired any additional amino acid changes in their HA or NA genes (data not shown). Thus, our results suggested that all introduced NA mutations remained genetically stable in A/Turkey/15/06 (H5N1) virus background, however, the possibility that some mutations in polymerase or other genes might occur during *in vitro* passaging cannot be excluded.

### NA Inhibitor Susceptibility and NA Enzyme Kinetics of Recombinant H5N1 Viruses

An NA enzyme inhibition assay, a reliable phenotypic assay used to characterize the NA inhibitor susceptibility of influenza viruses, showed altered susceptibility in all recombinant H5N1 influenza viruses except that with K150N substitution. The susceptibility of V116A, I117V, and N294S viruses to oseltamivir carboxylate or zanamivir was moderately reduced (mean IC_50_ increase, 5-63–fold and 3-33–fold, respectively) as compared to that of WT virus. Virus carrying the E119A mutation was moderately more resistant to oseltamivir carboxylate (35-fold increase in mean IC_50_ value) and markedly more resistant to zanamivir (>1200-fold increase in mean IC_50_ value) than was WT virus (Table 2). The H274Y mutant was much more resistant to oseltamivir carboxylate (mean IC_50_ increase, >900-fold) and slightly less susceptible to zanamivir (mean IC_50_ increase, 3-fold) than WT virus. In contrast, the Y252H NA change conferred increased susceptibility to oseltamivir carboxylate (mean IC_50_ decrease, 22-fold) (Table 2).

**Table 2:** Enzymatic Properties of the Neuraminidase of Recombinant H5N1 Influenza Viruses.

H5N1 recombinant virus	Mean IC_50_ ± SD (nM)^a^				Mean K*_m_* ± SD (µM)^b^ (95% CI)	V*_max_* ratio^c^	Mean K*_i_* ± SD (nM)^d^			
	Oseltamivir carboxylate	Mean fold change	Zanamivir	Mean fold change			Oseltamivir carboxylate	Mean fold change	Zanamivir	Mean fold change
**WT**	6.7±0.8	1.0	1.0±0.1	1.0	142.6±41.2 (49.4–235.7)	1.0	3.9±0.5	1.0	0.6±0.1	1.0
**V116A**	48.4±2.1*	7.2*	32.8±0.9*	32.8*	623.7±41.0* (531.0–716.5)	0.1*	41.6±1.8*	10.7*	28.2±0.8*	47.0*
**I117V**	34.3±1.6*	5.1*	4.1±0.3*	4.1*	742.0±139.5* (426.4–1057.0)	4.1*	30.2±6.7*	7.7*	3.6±0.3*	6.0*
**E119A**	236.5±55.9*	35.3*	1253.8±171.5*	1253.8*	1096.0±41.3* (1002.0–1189.0)	0.1*	216.7±51.3*	55.6*	1148.5±157.2*	1914.2*
**K150N**	8.3±1.2	1.2	1.2±0.1	1.2	110.4±15.5 (75.4–145.4)	0.1*	4.4±0.6	1.1	0.7±0.1	1.2
**Y252H**	0.3±0.1*	0.1*	1.3±0.1	1.3	199.9±62.3 (59.1–340.8)	1.8*	0.2±0.1*	0.1*	0.9±0.1	1.5
**H274Y**	6308.0±1199.5*	941.5*	2.5±0.1*	2.5*	130.9±10.5 (107.2–154.6)	0.1*	3573.5±905.8*	916.3*	1.4±0.1*	2.3*
**N294S**	424.2±13.5*	63.3*	3.4±0.3*	3.4*	298.5±15.1 (234.3–332.6)	0.1*	317.6±10.2*	81.4*	2.5±0.2*	4.2*

a NA inhibition assay was performed with viruses standardized to equivalent NA activity and incubated with NA inhibitors (0.00005–100 µM) and MUNANA substrate. IC_50_ was determined by plotting the dose-response curve of inhibition of NA activity as a function of the compound concentration. Values represent 3 independent determinations.

b K*_m_* represents a half-maximal catalytic rate. The enzyme kinetic data were fit to the Michaelis-Menten equation using GraphPad Prism 4. Values are the mean ± SD from one representative experiment done in triplicate.

c Ratio of the respective viruses’ NA V*_max_* to the V*_max_* of WT recombinant A/Turkey/15/06 virus NA. V*_max_* was calculated using a nonlinear regression of the curve according to the Michaelis-Menten equation.

d Determined by enzymatic kinetic analysis and calculated using nonlinear regression of the plot of initial velocity as a function of inhibitor concentration.

*P<0.01 compared to WT virus (one-way ANOVA).

**Figure 2:**
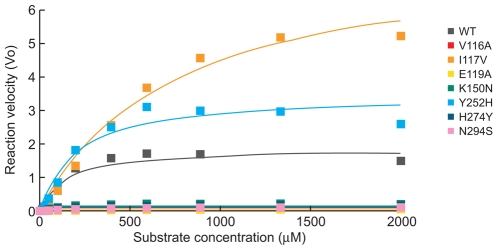
NA enzyme kinetics of the recombinant A/Turkey/15/06-like (H5N1) viruses. Substrate conversion velocity (V_0_) of NA is shown as a function of substrate concentration. Fluorogenic MUNANA substrate was used at a final concentration of 0 to 2000 µM. The viruses were standardized to an equivalent dose of 10^7.5^ PFU/ml. Fluorescence was measured every 92 sec for 45 min at 37°C, using excitation and emission wavelengths of 355 and 460 nm, respectively.

To determine the effect of the NA mutations on viral functional properties, we characterized their kinetic NA enzymatic parameters, including their Michaelis-Menten constants (K*_m_*), which reflect NA affinity for the MUNANA substrate, their inhibition constants (K*_i_*) for oseltamivir carboxylate and zanamivir, and their relative NA enzymatic activity (V*_max_*) (Table 2, Figure 2). NA proteins harboring the V116A, I117V, and E119A mutations exhibited significantly lower affinity for the substrate (mean K*_m_* decrease, 4–8–fold) than did other NAs studied. The K*_i_* values of all NA glycoproteins were consistent with their IC_50_ values (Table 2), suggesting that the reduced susceptibility to both NA inhibitors could be caused by decreased affinity of their mutant NAs to the antiviral drugs.

We also determined NA enzymatic V*_max_* values for each of the recombinant H5N1 viruses (Figure 2) and calculated their V*_max_* ratios in relation to that of WT NA glycoprotein (Table 2). NA proteins with mutations at residues 117 and 252 had significantly higher enzymatic activity than WT NA (*P*<0.01). The other five NA mutations studied significantly reduced the NA activity of A/Turkey/15/06 (H5N1) influenza virus (all V*_max_* ratios, 0.1) (Table 2, Figure 2). Thus, the eight recombinant H5N1 viruses differed in both their NA enzyme kinetics and their *in vitro* susceptibility to oseltamivir carboxylate and zanamivir.

### Pathogenicity of Recombinant H5N1 Viruses in Ferrets

**Figure 3:**
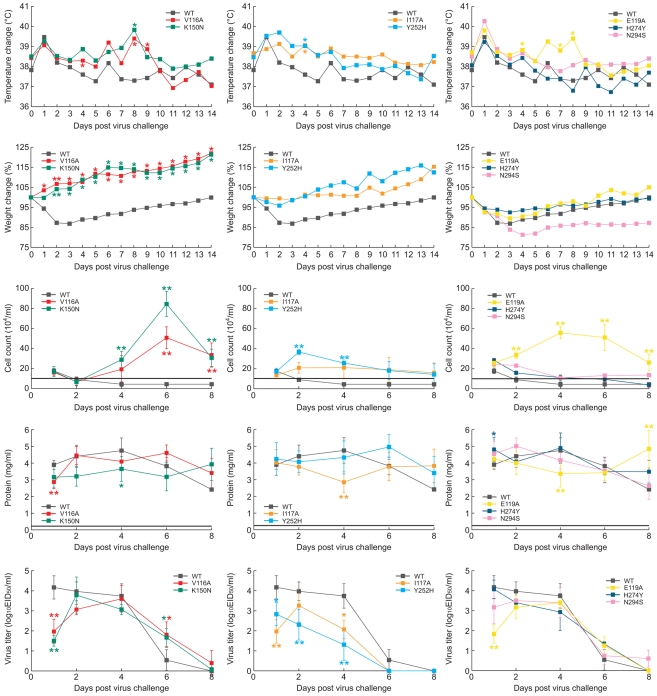
Patterns of clinical outcome in ferrets inoculated with the recombinant A/Turkey/15/06-like (H5N1) viruses. (A) Mild, prolonged illness, (B) mild, brief illness, and (C) severe illness or (comparable to WT) caused by the indicated recombinant viruses. Shown are change in body temperature and weight, total number of inflammatory cells and protein concentrations in nasal washes, and virus titers in the upper respiratory tract. The horizontal lines show the mean inflammatory cell counts and protein concentrations in the nasal washes of uninoculated animals. Values are the mean ± s.d. for three ferrets. The mean s.d. of all data points for change in body temperature and weight was ∼±4.5%. *, P<0.05, **, P<0.01 compared to WT virus (one-way ANOVA).

Limited information is available on the pathogenicity of H5N1 influenza viruses carrying various drug-resistant NA mutations in ferrets [23], although it can be an indicator of pathogenic potential of these viruses in humans. We inoculated ferrets with 10^6^ EID_50_ of each virus and observed four different patterns of clinical outcome (Table 3, Figure 3). Recombinant WT virus caused mild clinical disease signs of moderate duration, similar to those caused by wild-type A/Turkey/15/06 (H5N1) strain [10].

**Table 3:** Pathogenicity of the Recombinant H5N1 Influenza Viruses in Ferrets.

Reverse-genetics virus	Change in oseltamivir resistance^a^	No. of surviving/total no.	Mean survival time ± SD^b^	Relative inactivity index^c^	Mean serum antibody response^d^
*Mild, moderate duration*					
** WT**	1.0	3/3	>21.0	0.00	160
** H274Y**	941.5	3/3	>21.0	0.01	240
*Mild, prolonged duration*					
** V116A**	7.2	3/3	>21.0	0.00	80
** K150N**	1.2	3/3	>21.0	0.02	80
*Mild, short duration*					
** I117V**	5.1	3/3	>21.0	0.04	80
** Y252H**	0.1	3/3	>21.0	0.06	80
*Severe illness*					
** E119A**	35.3	2/3	15.3±0.3*	0.29*	80
** N294S**	63.3	2/3	17.7±0.3*	0.46*	240

a Mean fold-change in IC_50_ compared to that of WT virus.

b Determined by the Kaplan-Meier method (Mantel-Haenszel test).

c Determined once daily for 14 days of observation based on the scoring system described in Methods and was calculated as the mean score per group of ferrets per observation (day) over the 14-day period. The relative inactivity index before inoculation was 0.

d Hemagglutination inhibition (HI) titers determined in ferret sera 21 days p.i. with A/Turkey/15/06 (H5N1) virus. Data are from groups of 2 (for E119A and N294S viruses) or 3 ferrets.

*P<0.05 compared to WT virus (one-way ANOVA).

Mild but prolonged illness (duration ∼ 10 days, relative inactivity index, RII≈0.01) was seen in ferrets infected with mutant V116A and K150N viruses (Table 3). Despite their weight gain throughout the infection, the ferrets showed a mean peak temperature increase of ∼ 1.0–1.3°C on day 8 post-inoculation (p.i.) (Figure 3A). On days 4, 6, and 8 p.i. the peak nasal inflammatory cell counts in nasal washes was significantly higher in groups of ferrets inoculated with V116A and K150N recombinants than in those inoculated with WT virus (*P*<0.01). Virus shedding started at 1 day p.i. for mutant viruses and continued until 6 days p.i. Interestingly, on day 1 p.i. virus titers in nasal washes were significantly lower for animals infected with recombinant V116A and K150N viruses than those in animals inoculated with WT virus (*P*<0.05), but remained significantly higher on day 6 p.i. (*P*<0.05) (Figure 3A).

Mild, short illness (duration ∼ 5–6 days) was observed in ferrets inoculated with H5N1 viruses carrying I117V and Y252H NA mutations: only slight temperature elevation (mean peak increase, 0.5 to 1.2° C), mild clinical features (RII≈0.05) (Table 3) and minor weight changes were detected (Figure 3B). Cell counts remained at the same level in nasal washes of animals infected with either WT or I117V viruses, and they returned to normal levels in ferrets inoculated with Y252H at day 6 p.i. Comparison of the protein concentrations in the nasal washes showed no significant differences among the I117V, Y252H and WT viruses, suggesting that upper respiratory tract inflammation in these groups was comparable. Recombinant I117V and Y252H viruses replicated less efficiently in the upper respiratory tract than did the WT strain (*P*<0.05) (Figure 3B).

Virus with the H274Y NA mutation exhibited virulence comparable to that of recombinant WT virus (Table 3, Figure 3C). In contrast, inoculation of ferrets with recombinant E119A and N294S viruses caused markedly different results. Animals showed more pronounced clinical signs of disease, including a slightly greater RII (≈0.3–0.4), and one of three ferrets in each group was euthanized before the end of the experiment due to severe lethargy or excessive weight loss (Table 3). The body temperature elevation and nasal-wash inflammatory cell count were significantly higher in animals inoculated with the E119A mutant than in those inoculated with recombinant WT virus (*P*<0.01) (Figure 3C). The recovery of ferrets inoculated with the N294S NA mutant was delayed, and they regained no weight during the observation period.

To characterize in more details the disease caused by recombinant E119A and N294S viruses, we increased the number of animals from three to five in each of the pathogenicity groups and repeated inoculation of ferrets with 10^6^ EID_50_ of the WT, E119A, and N294S viruses (Figure S1). Importantly, all clinical symptoms of disease were similar to those observed in the first experiment (Figure 3C), including a slight increase in RII for the E119A and N294S (0.3 and 0.4, respectively) and substantial weight loss (∼15%) of N294S-infected ferrets. We observed that nasal-wash inflammatory cell counts were significantly higher in animals infected with mutant viruses than in those infected with WT (*P*<0.01) (Figure S1). Thus, in two independent experiments, the infection of ferrets with viruses carrying E119A or N294S NA mutations consistently caused more severe influenza disease than WT virus. Taken together, our results showed that changes in the framework residues of the NA enzyme active site can markedly affect the pathogenesis of clade 2.2 A/Turkey/15/06 (H5N1) influenza virus in ferrets.

### Histopathology and Replication of Recombinant WT, E119A, and N294S Viruses in Internal Organs of Ferrets

**Figure 4:**
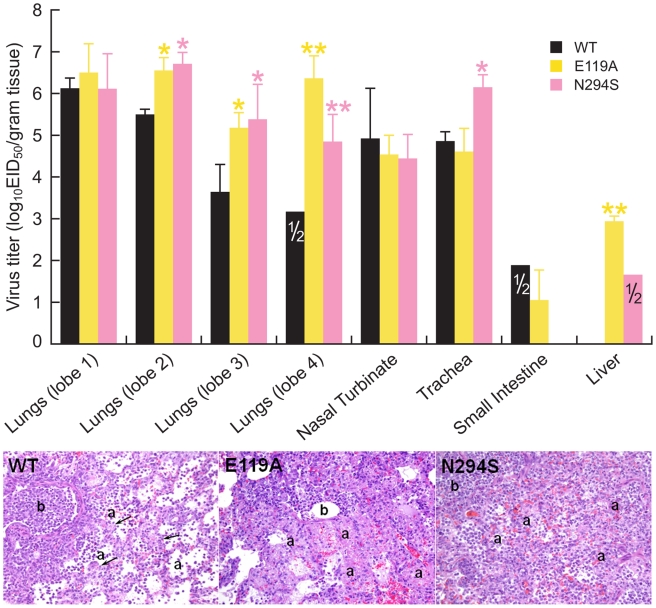
Replication of recombinant WT, E119A, and N294S viruses in internal organs and histologic changes in the lungs morphology of ferrets infected with these H5N1 viruses. (A) Virus titers were determined for the lungs (4 lobes tested separately), nasal turbinates, tracheas, small intestines, and livers of ferrets on day 4 post-inoculation. Values (log_10_EID_50_/gram tissue) are the mean ± s.d. for two ferrets, unless it is indicated that the virus was detected in one of two ferrets. *, P<0.05, **, P<0.01 compared to WT virus (one-way ANOVA). (B) The images shown are hematoxylin-and-eosin-stained sections of lung tissue from ferrets inoculated with the WT, E119A, and N294S viruses obtained on day 4 post-inoculation. Lung showing bronchiole (b) with epithelial necrosis, intraluminal debris, and inflammatory cells. Lung alveoli (a) of the WT-infected ferrets are lined with interstitial septa (arrows) and have mild to moderate inflammatory cell infiltrates. The lung alveolar architecture of the ferrets inoculated with the E119A mutant is lost due to necrosis of alveolar pneumocytes and the interstitial septa. Lung alveoli of the N294S-infected ferrets are filled with inflammatory cells and hyperplastic pneumocytes obscuring interstitial septa. Magnification, ×20.

To identify characteristics that may explain the severity of infection caused by E119A and N294S H5N1 mutants compared to WT stain, we evaluated virus replication and tissue tropism in the lungs, nasal turbinate, trachea, spleen, liver, and small intestine of two inoculated ferrets per virus on day 4 p.i. All three viruses were detected in the lungs, nasal turbinate, and trachea; however, only the E119A and N294S mutants were detected in the liver (Figure 4A). Consistent with the more pronounced clinical signs of disease, the E119A and N294S were detected at significantly higher titers in three out of four lobes of the lungs (∼4.9–6.7 log_10_EID_50_/gram tissue) and liver (∼1.7–2.9 log_10_EID_50_/gram tissue) (*P*<0.01). Of the three H5N1 viruses, the N294S recombinant yielded significantly higher virus titers in the trachea (∼6.2 log_10_EID_50_/gram tissue versus 4.7 log_10_EID_50_/gram tissue, *P*<0.01) (Figure 4A).

We further histologically examined the tissues of inoculated ferrets to investigate the lesions associated with virus replication in infected organs and to obtain more information about the differences in virulence between recombinant WT, E119A, and N294S viruses. Histological changes were detected only in the lungs of infected animals. Bronchopneumonia and bronchiolitis with epithelial necrosis and/or regeneration characterized by bronchiole hypertrophy and hyperplasia were observed in all ferrets on day 4 p.i. (Figure 4B). However, the degree of severity and the number of lung lobes showing pneumonic lesions differed between the three groups. We determined that only two lung lobes of both ferrets inoculated with WT virus had foci of bronchopneumonia. However, the ferrets inoculated with N294S recombinant had bronchopneumonia in ∼ 50% of two lung lobes and in >90% of the other two lobes. The E119A-infected animals revealed multifocal bronchopneumonia in >90% of all four lung lobes (Figure 4B).

Differences in the type and extent of the alveolar pathology were also apparent on histopathologic examination. Of the three H5N1 viruses, the E119A mutant caused very severe and extensive alveolitis with necrosis of the alveolar pneumocytes and interstitial septal walls resulting in the loss of the alveolar architecture and leading to large coalescing spaces of edema fluid, fibrin, inflammatory and hemorrhagic red blood cells (Figure 4B). In the N294S-infected ferrets, alveolitis was characterized by a centrifugal progression of severity from the peribronchiole alveoli to the subpleural alveoli with pneumocyte hyperplasia and extensive infiltrates of inflammatory cells obscuring the lace-like alveolar pattern. In contrast, in the WT group, the peribronchiole and peripheral alveoli had only mild to moderate inflammatory cell infiltrates and a lace-like pattern with a slight thickening of the alveolar interstitial septa (Figure 4B).

Additionally, to assay the genetic stability of the recombinant viruses *in vivo*, we extracted RNA directly from nasal wash samples obtained on day 6 p.i. and sequenced the NA and HA genes of the dominant virus populations. No amino acid substitutions were identified in the NA gene of viruses isolated from ferrets inoculated with the WT, E119A, or N294S recombinants. A single F291L mutation in the HA gene was identified in 1 of 3 ferrets inoculated with either E119A or N294S mutants.

Taken together, our results indicated that the introduced E119A and N294S NA mutations were stably maintained in A/Turkey/15/06 (H5N1) virus background in ferrets. Further, increased virulence and severity of disease caused by H5N1 viruses carrying E119A and N294S NA changes were associated with higher virus titers and more pronounced local inflammatory response in the lungs compared to WT virus.

## Discussion

Oseltamivir-resistant H1N1 influenza viruses emerged recently and became predominant during the 2007–2008 season in the absence of drug-selection pressure [27], [28]; however, the molecular mechanisms and viral characteristics underlying this phenomenon are unknown. The NA inhibitors are an important component of influenza pandemic preparedness. In the case of H5N1 influenza viruses, genetic diversity and continuous evolution [1], [2] are known to contribute to reduced susceptibility to NA inhibitors and thus to reduced drug efficacy. In the present study, we introduced single point NA mutations into the A/Turkey/15/06 (H5N1)-virus genetic background at framework and functional NA residues to assess their effect on the NA-inhibitor resistance phenotype and on their pathogenicity in a ferret model. Of 11 NA mutations studied, seven located within (E119A, H274Y, and N294S) or near (V116A, I117V, K150N, and Y252H) the enzyme active site were stably maintained and grew to titers comparable to WT virus in MDCK cells. Five of these 7 NA mutations (all but K150N and Y252H) reduced H5N1 virus susceptibility either to oseltamivir carboxylate or to both NA inhibitors.

Our most important finding is that the E119A NA substitution in the clade 2.2 A/Turkey/15/06 (H5N1)-virus background is viable and genetically stable and confers cross-resistance to oseltamivir carboxylate and zanamivir. Structural analysis showed that the glutamate at 119 is one of two conserved amino acid residues that interact with oseltamivir’s carboxylate group to allow a strong, specific bond between the enzyme active site and the inhibitor [29]. The amino acid alanine has shorter side chains than does glutamate, a difference that may impede this interaction. The resistance of the E119A NA mutant to both zanamivir and oseltamivir carboxylate suggests that they interact similarly with the conserved framework residues of viral NA [29]. E119G/A/D/V NA mutations are commonly reported to be associated with NA inhibitor resistance and were identified in influenza viruses of the N2 NA subtype [14], [15]. Further, the E119A NA substitution has been selected in several strains of influenza A (H4N2) and B viruses after *in vitro* passages in the presence of zanamivir and has resulted in reduced NA activity [30], [31]. In a previous study, the E119A mutant in an A/WSN/33 (H1N1)-virus background could not be rescued [32], suggesting that this mutation may impede the growth of H1N1 viruses more than that of H4N2 viruses. Moreover, the E119G NA mutation significantly compromised viral growth and was genetically unstable in a clade 1 A/Vietnam/1203/04 (H5N1)-virus background [24]. In contrast, we recently found that in mice, E119A mutation is stably maintained in the NA of clade 2 H5N1 virus during oseltamivir therapy and is associated with resistance to both NA inhibitors [13]. Our finding that E119A reduced the NA activity of H5N1 virus 10-fold without compromising viral yield in MDCK cells suggests that these changes in NA activity do not compromise the infectivity of these viruses due to their high replication ability.

In the present study, V116A and I117V mutants showed a low (∼6-fold greater than WT) level of resistance to oseltamivir carboxylate. This finding is consistent with those of Hurt et al [12], who indentified two H5N1 influenza isolates (A/Chicken/Indonesia/Wates/77/05 and A/Chicken/Vietnam/486A/04) that carried these NA mutations and had reduced NA inhibitor susceptibility. Conversely, A/WSN/33 (H1N1) virus with the I117V change was susceptible to both NA inhibitors [33]. This discrepancy in observed phenotype may be explained by usage of different N1 NA proteins of H1N1 and H5N1 viruses and/or to specific changes in different N1 antigens. Our study is the first to our knowledge to fully elucidate the role of the V116A and I117V NA amino acid substitutions in NA inhibitor resistance in a genetically homogeneous H5N1-virus background in the absence of concomitant HA and/or NA mutations. Importantly, residues 116 and 117 are fully conserved in group 1 NA glycoproteins (N1, N4, N5, and N8 [29]), and their location adjacent to R118 may affect one of the three arginine residues that bind the carboxylate of the substrate sialic acid with further effect on virus susceptibility to the anti-NA drugs [29].

We also confirmed previous findings that the presence of histidine at position 252 in recent H5N1 isolates is associated with increased affinity of their NAs for oseltamivir carboxylate [11], [18], [34]. In clade 1 NAs, this residue is normally H252, but in clade 2 NAs it is consistently Y252. Because this mutation increased the binding of A/Turkey/15/06 (H5N1) virus to oseltamivir, we speculate that genetic variation in highly pathogenic clade 2 H5N1 viruses in the absence of drug-selective pressure may reduce oseltamivir susceptibility *in vitro* (15- to 30-fold differences in IC_50_ values) [11] and *in vivo* [9].

We studied the pathogenicity and virulence of the NA-mutant H5N1 influenza viruses in ferrets, an acceptable animal model to evaluate influenza virus disease manifestations [35]. The inefficient transmissibility of H5N1 influenza viruses in these mammals [36] (as in humans) restricted our studies to evaluation of pathogenicity. Importantly, recombinant H5N1 viruses carrying framework NA mutations (E119A and N294S) were more virulent and were associated with significantly higher virus titers in the lungs and liver than WT virus (*P*<0.01). The molecular basis of this finding is unknown. However, the possibility that after exposure to one NA inhibitor the chance exists that occurring drug-resistant H5N1 mutant could be cross resistant to both antiviral drugs and will be more virulent that the WT virus (like E119A H5N1 recombinant virus) should prompt to re-assess the suitability of single-drug usage. None of the NA mutations studied resulted in decreased viral virulence in the ferret animal model, consistent with our group’s previous finding that NA inhibitor-resistant mutations did not impair the virulence of clade 1 A/Vietnam/1203/04 (H5N1) virus in mice [24]. Here we used A/Turkey/15/06 (H5N1) virus belonging to clade 2.2, whose virulence is lower than that of the A/Vietnam/1203/04 (H5N1) strain [10]. Its NA activity is lower as well but is higher than that of current seasonal human-like N1 NAs even in the presence of drug-resistant NA mutations (data not shown). Therefore, taken together, our results showed that the virulence of H5N1 influenza viruses does not seem to be decreased by the NA-inhibitor resistant mutations studied here. We did not observe a consistent pattern in the effect of enzyme kinetics (V*_max_* and K*_m_*) on virulence in ferrets. None of the recombinant mutants exhibited an increased affinity for the substrate and a higher activity compared to WT NA, which could possibly indicate their overall better fitness. However, recombinant H5N1 viruses carrying I117V and Y252H NA mutations possessed the highest relative NA activity, but ferrets infected with these viruses experienced a mild, brief illness. Additional studies are needed to identify the impact of NA activity and affinity on the duration of influenza disease *in vivo*.

Our data demonstrate the significance of continued characterization of all H5N1 isolates for susceptibility to NA inhibitors in order to identify novel NA markers of altered susceptibility. We believe that this knowledge is essential for planning appropriate management strategies for a future pandemic. It is noteworthy that alignment of the NA genes of currently circulating highly pathogenic H5N1 influenza A viruses identified the NA mutations studied here in ∼0.1%–1.4% of isolates (namely, I117V was found in 1.4% and V116A and I119A were found in 0.1% isolates), raising concern about the drug sensitivity of a possible pandemic strain. Future studies should focus on the establishment of novel antiviral strategies to minimize the emergence of resistance.

## Materials and Methods

### Ethics Statement

All animal experiments with recombinant H5N1 influenza viruses were performed in biosafety level 3+ facilities at St. Jude Children’s Research Hospital (St. Jude; Memphis, TN, USA). All animal studies were approved by the St. Jude Children’s Research Hospital Animal Care and Use Committee and were conducted according to applicable laws and guidelines.

### Cells, Viruses and Compounds

Madin-Darby canine kidney (MDCK) and human embryonic kidney (293T) cells were obtained from the American Type Culture Collection and maintained as previously described [24].

Eight plasmids were constructed from the DNA sequences of the 8 gene segments of wild-type A/Turkey/15/06 (H5N1) virus for the reverse-genetics generation of recombinant wild-type virus. Recombinant virus was generated by DNA transfection of 293T cells [37], and the point mutations (Table 1, Figure 1) were inserted into the NA gene of wild-type virus by using a Quickchange site-directed mutagenesis kit (Stratagene) [24]. Stock viruses were prepared in MDCK cells at 37°C for 72 h and their entire HA and NA genes were sequenced to verify the presence of the mutations. The recombinant viruses were designated according to their NA mutations (Table 1, Figure 1). All experimental work with the H5N1 recombinant viruses was performed in a biosafety level 3+ laboratory approved for use by the U.S. Department of Agriculture and the U.S. Centers for Disease Control and Prevention.

The NA inhibitors oseltamivir carboxylate (oseltamivir) ([*3R,4R,5S*]-4-acetamido-5-amino-3-[1-ethylpropoxy]-1-cyclohexene-1-carboxylic acid) and zanamivir (4-guanidino-Neu5Ac2en) were provided by Hoffmann-La Roche, Ltd.

### Stability and Infectivity of Recombinant H5N1 Viruses

The genetic stability of the viruses was monitored by sequencing of the HA and NA genes after transfection and after three passages in MDCK cells at a MOI of 0.001 PFU/ml. If different subpopulations were identified, those viruses were considered unstable.

The infectivity of recombinant H5N1 viruses was determined in MDCK cells by plaque assay and expressed as log_10_PFU/ml [9]. Briefly, confluent MDCK cells were incubated at 37°C for 1 h with 10-fold serial dilutions of virus. The cells were then washed and overlaid with minimal essential medium containing 0.3% bovine serum albumin and 0.9% Bacto agar and incubated at 37°C for 72 h. The plaques were stained with 0.1% crystal violet solution containing 10% formaldehyde, and virus yield was determined. Plaque diameter of any 10 plaques was measured by the Finescale® comparator (Los Angeles, CA, USA).

### NA Enzyme Activity and Kinetics

Modified fluorometric assay was used to determine the NA activity of the recombinant H5N1 viruses [38]. We measured the NA enzyme kinetics at pH 6.5 with 33 mM 2-(N-Morpholino)ethanesulfonic acid hydrate (MES; Sigma-Aldrich), 4 mM CaCl_2_, and fluorogenic substrate 2’-(4-methylumbelliferyl)-α-D-N-acetylneuraminic acid (MUNANA; Sigma-Aldrich; final substrate concentration, 0–2000 µM). All H5N1 viruses were standardized to an equivalent dose of 10^7.5^ PFU/ml. The reaction was conducted at 37°C in a total volume of 50 µl, and the fluorescence of released 4-methylumbelliferone was measured every 92 sec for 45 min in a Fluoroskan II instrument (Labsystems) using excitation and emission wavelengths of 355 and 460 nm, respectively. To measure the inhibitory effect of oseltamivir carboxylate or zanamivir on NA activity, H5N1 viruses were preincubated for 30 min at 37°C in the presence of various concentrations of the drugs (0.00005–5 µM). The kinetic parameters Michaelis-Menten constant (K*_m_*), maximum velocity of substrate conversion (V*_max_*), and inhibitory constant (K*_i_*) of the NAs were calculated by fitting the data to the appropriate Michaelis-Menten equations by using nonlinear regression in the commercially available GraphPad Prism 4 software (GraphPad Software, La Jolla, CA).

### NA Enzyme Inhibition Assay

Recombinant H5N1 viruses were standardized to equivalent NA activity and incubated for 30 min at 37°C with NA inhibitors at concentrations of 0.00005–100 µM with MUNANA (Sigma-Aldrich) as a substrate. After 1 h, the reaction was terminated by adding 14 mM NaOH and fluorescence was quantified in a Fluoroskan II (Labsystems) fluorometer. The concentration of NA inhibitor that reduced NA activity by 50% relative to a control mixture with no inhibitor (IC_50_) was determined by plotting the dose-response curve of inhibition of NA activity as a function of the compound concentration. Values are the mean of 2–3 independent determinations.

### Assessment of Virus Pathogenicity in Ferrets

Pathogenicity was tested in 4- to 5-month-old male ferrets obtained through the ferret breeding program at St. Jude Children’s Research Hospital or from Marshall Farms (North Rose, NY). All ferrets were seronegative for influenza A H1N1, H3N2, and H5N1, and for influenza B viruses. Groups of three ferrets were inoculated intranasally under light isoflurane anesthesia with 10^6^ EID_50_ of virus in 1 ml sterile phosphate-buffered saline (PBS). Clinical signs of infection, relative inactivity index [35], weight, and temperature were recorded daily. Activity level was assessed by using the following scoring system: 0, alert and playful; 1, alert but playful when stimulated; 2, alert but not playful when stimulated; and 3, neither alert nor playful when stimulated. The relative inactivity index was calculated as the mean score per group of ferrets per observation (day) over the 14-day period. Animals that showed signs of severe disease (respiratory signs [labored breezing, sneezing, wheezing, and nasal discharge], febrility, lethargy, steady weight loss, and neurological signs [hind-limb paresis, ataxia, torticollis, and tremor] and >25% weight loss were euthanized. Body temperature was measured by subcutaneous implantable temperature transponders (Bio Medic Data Systems Inc.).

To monitor virus shedding, nasal washes were collected from ferrets on days 1, 2, 4, 6, and 8 post-challenge. Virus was titrated in 10-day old embryonated chicken eggs by injecting 0.1 ml of serial 10-fold dilutions of the sample (three eggs per dilution) and expressed as log_10_EID_50_/ml. Inflammatory cell counts and protein concentrations in nasal washes were determined as described previously [39]. Briefly, the nasal washes were centrifuged at 2,000 rpm for 10 min. The cell pellet was resuspended in PBS, and the cells were counted microscopically in a hemacytometer. The total number of inflammatory cells was calculated on the basis of the initial volume of nasal wash. The protein concentration in cell-free nasal washes was measured by using a protein reagent from Bio-Rad (Hercules).

### Titration of Virus in Organs

Two animals inoculated with WT, E119A, or N294S viruses were euthanized by intracardiac injection of Euthanasia V solution on day 4 post-inoculation, and tissue samples (∼0.5 g each) were collected from lungs (4 lobes tested separately), nasal turbinate, trachea, spleen, liver, and small intestine. Samples were homogenized in 1 ml sterile PBS with antibiotics, and the virus titer (log_10_EID_50_/gram tissue) was determined in 10-day old embryonated chicken eggs.

### Histopathologic Analysis

Tissues (lung, nasal turbinate, trachea, spleen, liver, and small intestine) were collected at the time of necropsy, fixed in 10% neutral-buffered formalin, and embedded in paraffin. Five-micrometer-thick sections were stained with hematoxylin and eosin and studied by light microscopy.

### Serologic Tests

Serum samples were collected from ferrets 3 weeks after inoculation, treated with receptor-destroying enzyme, heat-inactivated at 56 °C for 30 min, and tested by hemagglutination inhibition (HI) assay with 0.5% packed chicken red blood cells by a standard method as described previously [40].

### Virus Sequence Analysis

Viral RNAs were isolated from virus-containing cell culture fluid after transfection, after three passages in MDCK cells or from ferret nasal washes by using the RNeasy Mini kit (Qiagen). Samples were reverse-transcribed and analyzed by PCR using universal primers specific for the HA and NA gene segments, as described previously [41]. Sequencing was performed by the Hartwell Center for Bioinformatics and Biotechnology at St. Jude. The DNA template was sequenced by using rhodamine or dRhodamine dye terminator cycle-sequencing Ready Reaction kits with AmpliTaq DNA polymerase FS (Perkin-Elmer) and synthetic oligonucleotides. Samples were analyzed in a Perkin-Elmer Applied Biosystems DNA sequencer (model 373 or 377). DNA sequences were completed and edited by using the Lasergene sequence analysis software package (DNASTAR).

### Statistical Analysis

The virus yield, plaque size, NA inhibitor susceptibility, NA enzyme kinetic parameters (K*_m_*, V*_max_*, K*_i_*), virus titers in ferret organs and nasal wash samples, differences in fevers, weights, total number of cells and protein concentrations in nasal wash samples, mean survival time, and relative inactivity index of ferrets inoculated with wild-type and mutant viruses were compared by analysis of variance (ANOVA). Virus yields and plaque size of recombinant H5N1 viruses after the first and the third passage in MDCK cells were compared by unpaired two-tailed *t-*test. The probability of survival was estimated by the Kaplan-Meier method and compared between groups of ferrets by using the log-rank (Mantel-Haenszel) test [42]. A probability value of 0.05 was prospectively chosen to indicate that the findings were not the result of chance alone.

## Supporting Information

Figure S1 Patterns of clinical outcome in ferrets inoculated with recombinant WT, E119A and N294S viruses. Shown are change in body temperature and weight, total number of inflammatory cells and protein concentrations in nasal washes, and virus titers in the upper respiratory tract. The horizontal lines show the mean inflammatory cell counts and protein concentrations in the nasal washes of uninoculated animals. Values are the mean ± s.d. for five (or three on days 6 and 8 p.i.) ferrets. The mean s.d. of all data points for change in body temperature and weight was ∼±4.5%. *, P < 0.05, **, P < 0.01 compared to WT virus (one-way ANOVA).
(0.48 MB TIF)
