# Supplementary figures and images for: Effect of Neuraminidase Inhibitor–Resistant Mutations on Pathogenicity of Clade 2.2 A/Turkey/15/06 (H5N1) Influenza Virus in Ferrets
Source: PLoS Pathog. 2010 May 27;6(5):e1000933. doi: 10.1371/journal.ppat.1000933 (PMC2877746; doi:10.1371/journal.ppat.1000933)

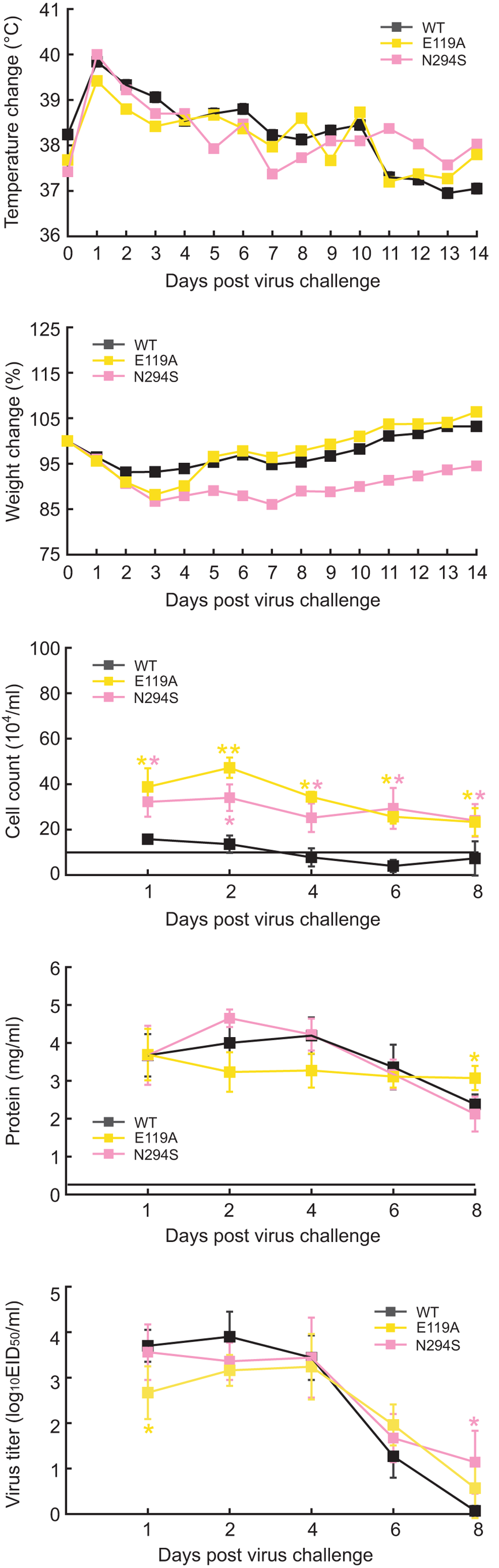

Supplement: Figure S1 — Patterns of clinical outcome in ferrets inoculated with recombinant WT, E119A and N294S viruses. Shown are change in body temperature and weight, total number of inflammatory cells and protein concentrations in nasal washes, and virus titers in the upper respiratory tract. The horizontal lines show the mean inflammatory cell counts and protein concentrations in the nasal washes of uninoculated animals. Values are the mean ± s.d. for five (or three on days 6 and 8 p.i.) ferrets. The mean s.d. of all data points for change in body temperature and weight was ∼±4.5%. *, P < 0.05, **, P < 0.01 compared to WT virus (one-way ANOVA). (0.48 MB TIF) [file ppat.1000933.s001.tif]
